# Efficacy of a Crosslinked Hyaluronic Acid-Based Hydrogel as a Tear Film Supplement: A Masked Controlled Study

**DOI:** 10.1371/journal.pone.0099766

**Published:** 2014-06-10

**Authors:** David L. Williams, Brenda K. Mann

**Affiliations:** 1 Department of Veterinary Medicine, Cambridge University, Cambridge, United Kingdom; 2 SentrX Animal Care, Inc., Salt Lake City, Utah, United States of America; 3 Department of Bioengineering, University of Utah, Salt Lake City, Utah, United States of America; 1Biomaterials for Regenerative Therapies Group, Institute for Bioengineering of Catalonia, Baldiri Reixac 15-21, Barcelona 08028, Spain, 2Technical University of Catalonia, Av. Diagonal 647, Barcelona 08028, Spain, 3CIBER-BBN, María de Luna 11, Zaragoza 50, Spain

## Abstract

Keratoconjunctivitis sicca (KCS), or dry eye, is a significant medical problem in both humans and dogs. Treating KCS often requires the daily application of more than one type of eye drop in order to both stimulate tear prodcution and provide a tear supplement to increase hydration and lubrication. A previous study demonstrated the potential for a crosslinked hyaluronic acid-based hydrogel (xCMHA-S) to reduce the clinical signs associated with KCS in dogs while using a reduced dosing regimen of only twice-daily administration. The present study extended those results by comparing the use of the xCMHA-S to a standard HA-containing tear supplement in a masked, randomized clinical study in dogs with a clinical diagnosis of KCS. The xCMHA-S was found to significantly improve ocular surface health (conjunctival hyperaemia, ocular irritation, and ocular discharge) to a greater degree than the alternative tear supplement (*P* = 0.0003). Further, owners reported the xCMHA-S treatment as being more highly effective than the alternative tear supplement (*P* = 0.0024). These results further demonstrate the efficacy of the xCMHA-S in reducing the clinical signs associated with KCS, thereby improving patient health and owner happiness.

## Introduction

Dry eye or keratoconjunctivitis sicca (KCS), is a widespread problem in both the human and canine populations. The prevalence of KCS in humans may vary between 5 and 33%, in different reports and with different methods of ocular evaluation [1]. The prevalence in the canine species varies between 1 to 4% [2]. Topical cyclosporine has been developed as a widely efficacious lacrimogenic agent in dogs [3] and more recently in man, [4] but not all individuals in either affected population respond adequately to the drug by a higher rate of tear production. Also, the high price of the product puts it out of the financial reach of many dog owners. For these reasons an effective, less expensive tear replacement eyedrop is still required. Many of these are available as topical medications containing a wide number of lubricating agents, including polyacrylic acid, polyvinyl alcohol, and hyaluronic acid (HA) [2], [5]. Since HA is a naturally occurring polysaccharide found as a lubricative agent in joint fluid, its use as a similar agent on the ocular surface is particularly appropriate [6], [7]. For tear supplements containg HA, previous reports have shown that the viscoelasticity of the polysaccharide leads to an increase in tear stability and a consequent reduction in many of the symptoms of dry eye [8]–[10].

The viscoelasticity of HA-based products can vary significantly, depending on the molecular weight and concentration of the HA used, as well as the concentration of salts present due to interaction with the polyanionic HA [8], [11], [12]. Such variation in rheologic properties, such as viscoelasticity, can lead to differences in comfort and efficacy for a dry eye formulation [13]. Typical HA-based tear supplements have been a simple solution of high molecular weight, low concentration HA. However, by covalently crosslinking HA, such as the formulation documented herein, leads to a more viscoelastic material. This increase in viscoelasticity extends the contact time of the HA with the ocular surface and will thus allow for less frequent application, reducing the overall cost and burden on the patient, and in the case of dogs, the owner. The covalent HA crosslinking described here, acts in a different manner than the physical or ionic crosslinking occurring in solutions of simple high molecular weight HA.

The crosslinked modified HA, thiolated carboxymethyl HA (CMHA-S), used in the present study has previously been used in other formulations to treat skin and corneal wounds [14], [15]. The hydrogel formulation used in this study was specifically developed as a tear supplement for the treatment of canine KCS. We have previously characterised the hydrogel rheologically to compare with non-crosslinked solutions of HA [16]. We also compared the ocular surface effects of this product to a previous study using a different tear replacement drop, evaluating tear production by use of the Schirmer tear test, conjunctival hyperaemia, ocular discharge and ocular irritation as determined by blink frequency and palpebral apperture narrowing [17]. Although the previous study demonstrated promising results, it was neither masked nor randomised, and the comparison of the products relied on two populations of KCS-affected dogs. Here we present the results of a study in which KCS-affected dogs were randomly assigned to treatment with either the CMHA-S product or a commercial tear replacement drop. Importantly, the medication was dispensed in such a manner that the investigator could not know which medication was being provided. Only after completion of the study and all assessments made following medication was the treatment regime for each dog unmasked, thus allowing a truly masked study. The statistical analyses of the data were also blinded in that statisticians were provided only “treatment one” and “treatment two” identification for each dog.

## Materials and Methods

### Crosslinked CMHA-S hydrogel

CMHA-S was synthesized and analyzed as previously described [15], [16]. A CMHA-S solution was then filter-sterilized, crosslinked to form a hydrogel, and packaged aseptically into sterile 10-ml eye drop bottles as previously described [16].

### Animals

The study was reviewed and accepted by the Ethics and Welfare Committee of the Department of Veterinary Medicine, University of Cambridge, Cambridge, UK and all animals were treated in accordance with the welfare guidelines in the Royal College of Veterinary Surgeons Guide to Professional Conduct.

Twenty dogs affected with KCS (as diagnosed clinically) and for whom treatment with topical cyclosporine (Optimmune, Schering-Plough UK) was either ineffective or not available for financial reasons were entered into the study with full informed owner consent. The gender, breed, and age of the dogs in the study is given in Table 1.

**Table 1:** Demographics and composite pre-treatment and post-treatment scores of ocular health for xCMHA-S and ITRD treatment of 10 dogs each.

Dog ID#	Breed	Age	Sex/neuter	Pre-score	Post-score
***xCMHA-S Treatment***					
601	Boxer	12	fn	6	1
606	WHWT	6	fn	14	5
608	JRT	11	fe	4	2
609	WHWT	12	fn	9	0
610	CKCS	9	fn	6	0
611	Shih Tzu	8	mn	4	0
614	ECS	12	fn	15	2
615	ACS	9	me	9	0
617	CKCS	10	fn	10	4
618	WHWT	12	fn	11	2
***ITRD Treatment***					
602	WHWT	7	fn	11	6
603	Lhasa Apso	8	me	6	3
604	CKCS	5	mn	9	4
605	CKCS	7	fn	9	3
607	X-bred	8	fn	8	3
612	X-bred	12	me	12	6
613	Labrador	10	fn	6	3
616	Lhasa Apso	8	fe	13	8
619	Cairn terrier	14	fe	14	6
620	ECS	9	mn	10	7

In Breed: WHWT  =  West Highland white terrier; JRT  =  Jack Russell terrier; CKCS  =  Cavalier King Charles spaniel; ECS  =  English cocker spaniel; ACS  =  American cocker spaniel; X-bred  =  mixed breed. In Gender: Fn  =  neutered female; Fe  =  unaltered female; Mn  =  neutered male; Me  =  unaltered male.

### Clinical evaluation and treatment

All dogs underwent full clinical and ophthalmic examination using a direct and indirect ophthalmoscopy and slit lamp biomicroscopy. Tear production was measured using the Schirmer tear test and ocular surface health assessed with the clinical measurements of conjunctival hyperaemia, ocular discharge and ocular irritation, graded as absent (0), mild (1), moderate (2) or severe (3). Tear supplement, either the crosslinked modified HA product (xCMHA-S) or the HA-based iDrop® Vet Plus Eye Lubricant (ITRD; I-MED Animal Health), was dispensed without the investigator being made aware of the treatment given. Dogs were treated for three weeks before reassessment. Owners were requested to use the trial medication alone three times daily. On re-examination a full ophthalmic examination was undertaken with conjunctival hyperaemia, ocular discharge and ocular irritation assessed and graded as previously. Owners were asked for their own subjective assessment of whether the treatment given was effective in ameliorating their animal's ocular symptoms, rating this from not effective (0) to highly effective (3).

The number of dogs included in the study was determined by a power analysis [18]. Using a desired effect size (the difference in mean between the treatment groups) for the composite score of 2.5, a standard deviation of 1.9 (based on the previous prospective study [16]), a type I error of 0.05, and power of 0.8, a sample size needed for each treatment group was 10.

### Statistical analysis

The primary outcome was defined as the composite post-treatment score, as the sum of the six post-treatment scores for each dog (hyperaemia, irritation, and discharge for each eye). This score was compared with the composite pre-treatment score using an analysis of covariance (ANCOVA) with the post-treatment score as the dependent variable, treatment as the main effect and pre-treatment score as the covariate. The pre-treatment score was used as a covariate because dogs that started with low scores cannot improve to the same extent as can dogs starting with higher scores, and thus improvement depends to some degree on the pre-treatment scores. A two-tailed t-test was used to compare the average age of dogs in the two groups, as well as owner happiness after treatment for the two groups. As previously mentioned, statistical analyses were made with the statistician blinded to the treatment given.

## Results

The treatment provided to each dog is shown in Table 1 together with composite pre-treatment and post-treatment scores. Dogs of a range of species were included with breeds predisposed to KCS such as West Highland White terriers, Lhasa Apsos, Shih Tzus, Cocker Spaniels and Cavalier King Charles spaniels predominating. The genders of the dogs were 7 female neutered animals, one entire bitch, one entire dog and one neutered dog in the xCHMA-S arm and 4 female neutered animals, two entire bitches, two entire dogs and one neutered dog in the ITRD arm of the study. The average age of dogs in the xCHMA-S arm (10.1±2.1 years) was not significantly different than in the ITRD arm (8.8±2.6 years) (*P* = 0.23).

**Figure 1:**
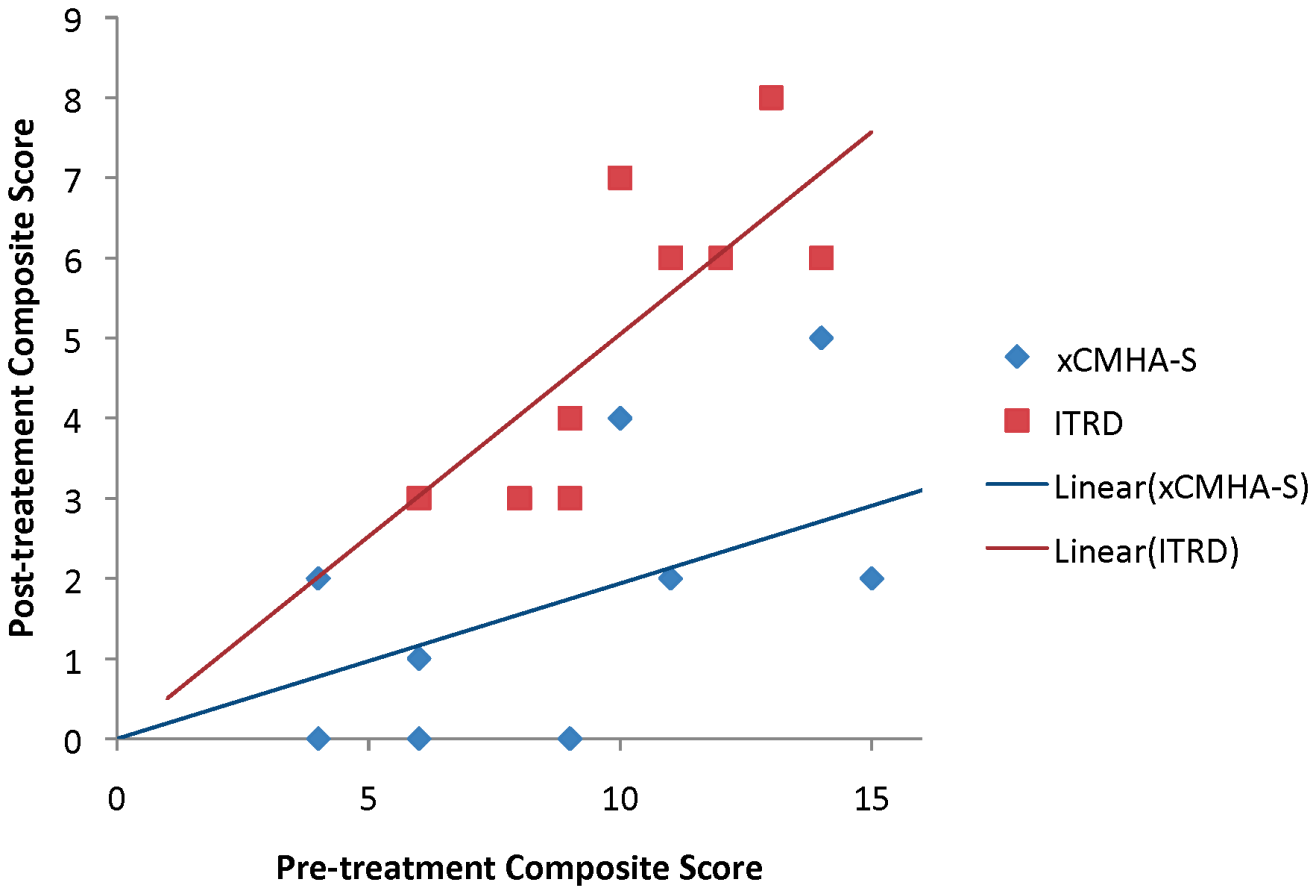
Composite score results. Post-treatment composite score plotted against pre-treatment composite score for xCMHA-S and ITRD treatments for all 20 dogs in the study. Note that for xCMHA-S, 2 dogs had a pre-treatment composite of 9 and post-treatment composite of 0; for ITRD, 2 dogs had a pre-treatment composite of 6 and post-treatment composite of 3. Lines indicate linear fits for each treatment.

Pre-treatment and post-treatment scores for STT, hyperaemia, irritation, and discharge for each dog are provided in Table 2, along with the owner happiness ratings at the end of the treatment period. Figure 1 shows the difference between pre-treatment and post-treatment composite scores for all dogs, demonstrating that xCHMA-S gives a substantially better resolution of KCS-associated ocular surface signs than does ITRD. The linear fits are forced through the origin, as no improvement is expected for cases with pre-scores of 0. For both treatments, the trends of the post-treatment scores are roughly proportional to the pre-treatment scores. There was no significant increase in STT following treatment with either xCMHA-S or ITRD (right eye: *P* = 0.1773 for both treatments; left eye: *P* = 0.5086 for xCMHA-S, 0.2695 for ITRD). Additionally, there was no significant difference between treatment groups for post-treatment STT, using pre-treatment STT as a covariate (right eye: *P* = 0.9445; left eye: *P* = 0.6170).

**Table 2:** STT values and scores of ocular health pre- and post-treatment, and owner happiness post-treatment with xCMHA-S or ITRD.

Dog ID#	STT pre (mm/min)	STT post (mm/min)	Hyperaemia pre	Hyperaemia post	Irritation pre	Irritation post	Discharge pre	Discharge post	Owner happiness
***xCMHA-S Treatment***									
601	3/11	5/12	3/0	1/0	2/0	0/0	1/0	0/0	3
606	0/1	0/2	3/3	1/0	2/2	1/1	3/1	1/1	3
608	5/5	6/4	2/2	1/0	0/0	1/0	0/0	0/0	2
609	6/4	6/5	2/2	0/0	2/1	0/0	1/1	0/0	3
610	7/5	6/6	2/1	0/0	2/1	0/0	0/0	0/0	3
611	8/9	10/8	1/1	0/0	0/0	0/0	1/1	0/0	2
614	0/0	0/0	3/3	0/1	2/2	0/0	3/2	1/0	3
615	3/3	2/2	2/2	0/0	1/1	0/0	2/1	0/0	3
617	4/3	5/4	2/2	0/0	2/2	1/1	1/1	1/1	3
618	0/1	1/1	2/2	0/0	2/2	0/0	2/1	1/1	3
Mean ± SD	3.60±2.95/4.20±3.52	4.10±3.25/4.40±3.60	2.20±0.63/1.80±0.92	0.30±0.48/0.10±0.32	1.50±0.85/1.10±0.88	0.30±0.48/0.20±0.42	1.40±1.07/0.80±0.63	0.40±0.52/0.30±0.48	2.80±0.42
***ITRD Treatment***									
602	1/1	3/1	3/3	1/1	1/1	1/1	1/2	1/1	1
603	3/4	5/4	1/1	0/1	2/1	1/1	1/0	0/0	2
604	5/3	6/2	1/2	1/1	2/2	1/1	1/1	0/0	1
605	4/4	3/5	2/2	1/1	2/1	0/1	1/1	0/0	2
607	4/4	4/5	1/1	0/0	1/1	0/1	2/2	1/1	2
612	0/0	0/0	3/3	1/1	1/1	1/1	2/2	1/1	3
613	0/16	0/18	3/0	1/0	3/0	1/0	0/0	1/0	3
616	2/1	1/3	3/3	2/2	1/2	1/1	2/2	1/1	1
619	2/2	3/2	3/3	1/1	2/2	1/1	2/2	1/1	2
620	4/3	5/2	2/2	1/1	2/2	2/1	1/1	1/1	1
Mean ± SD	2.50±1.78/3.80±4.52	3.00±2.11/4.20±5.12	2.20±0.92/2.00±1.05	0.90±0.57/0.90±0.57	1.70±0.67/1.30±0.67	0.90±0.57/0.90±0.32	1.30±0.67/1.30±0.82	0.70±0.48/0.60±0.52	1.80±0.79

STT  =  Schirmer tear test; values given are for right eye/left eye. Scores given for hyperaemia, irritation, and discharge are for right eye/left eye and indicate: absent (0), mild (1), moderate (2), or severe (3). Owner happiness scores were rated from not effective (0) to highly effective (3).

Table 3 provides the ANCOVA p-value for composite scores, showing that the coefficient for treatment, adjusted for pre-treatment scores, is highly significant (*P* = 0.0003). These results indicate that xCHMA-S treatment significantly improved composite ocular surface health compared to ITRD treatment. The adjustment for pre-score was also highly significant (*P* = 0.0018). The effects of age, sex, and neuter status were also tested, but were not found to be significant (*P* = 0.4405, 0.6298, and 0.3841, respectively). Additionally, a test of the slopes in Figure 1 indicated that they were highly significantly different (*P* = 0.00006). The post-scores for ITRD treatment were higher than the post-scores for xCMHA-S treatment, despite accounting for differences in pre-scores between the two treatments.

**Table 3:** Results of ANCOVAs for composite scores and individual assessments.

Assessment	P-value
Composite score	0.0003
Conjunctival hyperaemia (right)	0.0060
Conjunctival hyperaemia (left)	0.0003
Degree of irritation (right)	0.0261
Degree of irritation (left)	0.0002
Amount of discharge (right)	0.1094
Amount of discharge (left)	0.6093

The p-value provided compares the xCMHA-S treatment to the ITRD treatment, with post-treatment score as the dependent variable, treatment as the main effect, and pre-treatment score as the covariate.

ANCOVA analysis was performed on each of the six assessment criteria as well, and the resulting *P*-values are also shown in Table 3. Conjunctival hyperaemia and degree of irritation show statistically significantly greater improvement with xCMHA-S treatment than ITRD treatment, although the degree of irritation result for the right eye is not significant with the Bonferroni adjustment for multiple testing. The amount of discharge was not significantly different, likely because it is a much more variable clinical sign than conjunctival hyperaemia or ocular irritation. In fact, several dogs had low discharge scores pre-treatment in both groups, leading to this non-significant difference. The eyes are shown separately since conflating the results in eyes in which clinical signs are likely to be correlated would give inappropriately elevated degrees of significance [19].

Owner happiness with the results of treatment was significantly higher with xCHMA-S treatment than with ITRD (*P* = 0.0024). For xCMHA-S treatment, the average score was 2.8±0.4, with eight of the 10 owners rating the treatment as highly effective (score of 3). For ITRD treatment, the average score was 1.8±0.8, with only two of the 10 owners rating the treatment as highly effective.

## Discussion

Treatment for dry eye (KCS) can be taxing, whether in canine patients or humans. The advent of topical cyclosporine has substantially improved the lot of individuals in which this treatment is effective, but there are dogs in which the medication does not have the desired lacrimomimetic effects and for many owners the drug is too expensive, given as it must be used for the lifetime of the animal. Yet regular treatment with topical tear replacers can be difficult for owner and pet, and for human patients also.

In a previous study we reported the use of a crosslinked HA-based hydrogel as a tear supplement in a clinical study of 25 dogs with KCS [16]. Although the study demonstrated the potential for the xCMHA-S to reduce the clinical signs associated with KCS, the study was not masked or randomized. Thus, here we conducted a masked, randomized study comparing the xCMHA-S hydrogel and a standard tear replacement eye drop containing HA.

The results show a statistically significantly better therapeutic efficacy, based on ocular surface health, with the xCMHA-S gel applied three times daily than with the standard tear replacement drop. ANCOVA analysis was necessary here, with the pre-treatment score as a covariate, since the pre-treatment score will affect the degree to which the score can improve. Because the pre-treatment score estimate is not equal to 1.0, it underlines the idea that the treatment cannot have the same effect if a component of the composite pre-treatment score is coded as 0 as it can if that component is coded 3.

Although ocular surface health was improved, there was no significant improvement in STT value with either treatment. This was expected since both types of drops are merely tear supplements and do not stimulate the production of tears [16]. Additionally, owners were happier with the outcome of using the xCMHA-S gel compared to the standard tear replacement drop. Since this clinical study involved canine patients, it was not possible to assess any potential disruption in vision due to the increased viscosity of the xCMHA-S gel compared to the ITRD drops. However, if the owners had observed any vision issues, it is likely that the owner happiness scores would have been lower.

These findings have important implications for the canine population where an effective ocular surface lubricant will be welcomed by owners and canine patients alike. This crosslinked CMHA-S hydrogel may have potential translational importance as well, as an effective tear replacement drop with a long ocular surface residence time and thus a low required dose frequency would be highly valuable for the sizeable population of humans with dry eye.
